# Effect of oophorosalpingo-hysterectomy on serum antioxidant enzymes in female dogs

**DOI:** 10.1038/s41598-019-46204-w

**Published:** 2019-07-04

**Authors:** Linaloe Guadalupe Manzano Pech, Sara del Carmen Caballero-Chacón, Verónica Guarner-Lans, Eulises Díaz-Díaz, Adrián Moreno Gómez, Israel Pérez-Torres

**Affiliations:** 1 Department of Pathology, Instituto Nacional de Cardiología “Ignacio Chávez”, Juan Badiano 1, Sección XVI, Tlalpan, 14080 Mexico City, Mexico; 2 Department of Physiology and Pharmacology, Facultad de Medicina Veterinaria y Zootecnia, UNAM. Delfin Madrigal, Coyoacán, 04510 Mexico City, Mexico; 3 Department of Physiology, Instituto Nacional de Cardiología “Ignacio Chávez”, Juan Badiano 1, Sección XVI, Tlalpan, 14080 Mexico City, Mexico; 4 Department of Reproductive Biology, Instituto Nacional de Ciencias Medicas y Nutrición “Salvador Zubirán”, Vasco de Quiroga 15, Sección XVI, Tlalpan, 14000 Mexico, DF Mexico

**Keywords:** Obesity, Menopause

## Abstract

There are few studies evaluating the oxidant-antioxidant status after oophorosalpingohysterectomy (OSH) in female dogs. Here we determined the effect of OSH on antioxidant enzymes in serum, and quantified morphological changes in subcutaneous adipocytes. Lateral OSH was performed in 12 female dogs. The concentration of 17β-estradiol (17β-E_2_), the activities of extracellular superoxide dismutase (SOD-ec), glutathione peroxidase (GPx), glutathione-S-transferase (GST) and glutathione reductase (GR) were determined. Glutathione (GSH), glutathione disulfide (GSSG), lipid peroxidation (LPO), total antioxidant capacity (TAC), carbonylation and vitamin C were measured in serum. Subcutaneous adipose tissue was obtained to determine morphological changes and cell number, under basal conditions and six months after OSH. The SOD-ec, GPx and GST activities increased significantly (p ≤ 0.05), LPO, carbonylation and GSSG also increased. GSH and vitamin C decreased (p = 0.03). 17β-E_2_ tended to decrease six months after OSH. Hypertrophy of subcutaneous adipocytes was observed after OSH from the first month and was accentuated after six months (p = 0.001). The results suggest that 17β-E_2_ decreases after OSH and alters the antioxidant enzyme activities in serum thus, redox balance is altered. These changes are associated with an increase in body weight and hypertrophy of subcutaneous adipose tissue.

## Introduction

Oxidative stress (OS) is the consequence of an imbalance between the production of reactive oxygen species (ROS) and the total antioxidant capacity (TAC) of the organism. OS contributes to trigger inflammatory processes and endothelial dysfunction which is important in the development of cardiovascular diseases^1^. ROS are molecules or atoms that contain an unpaired electron in their last external orbital, acquiring a very unstable configuration^2^. The defense systems against ROS include; preventive mechanisms, reparatory mechanisms, physical defenses and antioxidant defenses. The antioxidant enzymatic defense mechanisms include superoxide dismutase (SOD), glutathione peroxidase (GPx) and catalase (CAT). Ascorbic acid (vitamin C), alpha tocopherol (vitamin E), glutathione (GSH), carotenoids, flavonoids, among other molecules, constitutes the non-enzymatic antioxidants^3^.

Superoxide dismutases (SODs) are metallic enzymes, whose function is to catalyze the reduction of super oxide (O_2_^−^) to molecular oxygen (O_2_) and hydrogen peroxide (H_2_O_2_). They are classified according to the different metallic cofactors they use, being divided into copper/zinc (Cu/Zn), manganese (Mn) and extracellular fluids (ec) SODs^4^.

Glutathione peroxidase (GPx) uses selenium as a cofactor; its function is to catalyze the reaction by which GSH reacts with peroxides to convert it into water. In this process, GSH is oxidized to glutathione disulfide (GSSG), and then reduced to GSH, by the enzyme glutathione reductase (GR)^3^.

Glutathione (GSH) is a tripeptide consisting of the amino acids glutamic acid, glycine and cysteine. It protects the cells against oxidative damage to lipids, proteins and nucleic acids. When ROS are present, GSH is oxidized to GSSG^4^. It has a synergistic interaction with other antioxidants such as vitamin C and E. GSH also acts by trapping OH• and O_2_^−^ radicals and it reactivates some enzymes that are involved in its detoxification. GSH is synthesized in the cytosol by the sequential action of glutamate-cysteine synthase and GSH synthetase^4^.

The water- soluble molecule of vitamin C, removes ROS from the hydrophilic compartments of the cell, extracellular matrix and the circulatory system. It also has the capacity to regenerate vitamin E^4^.

Estrogens, such as 17 beta estradiol (17β-E_2_), constitute the main product of ovarian granulose cells and are responsible for the production of other sexual steroids such as androstenedione and testosterone (T)^5^. 17β-E_2_ participates in the oxidative balance by promoting and/or preventing the production of ROS in mitochondria. It acts as a messenger that transmits a signal that activates the union of three transcription factors sensitive to oxidants^6^. The antioxidant and ROS eliminating activity of 17β-E_2_ is independent of receptor binding. It is associated with its chemical structure which has an intact OH group in the phenolic A ring of the molecule^7^. Other sex hormones which lack this ring, such as T, lack antioxidant effects^8^. Despite studies on the antioxidant effect of 17β-E_2_, the mechanisms of action and the structural conditions met by this hormone are not fully understood^9^. For example, 17β-E_2_ elevates GSH levels in neuronal and glial cells of the central nervous system and helps maintaining the redox-reduction state having activity against ROS^10^. Paradoxical reactions have also been observed in different species; for example, 17β-E_2,_ administration in ovariectomized (Ovx) Wistar rats reduces catalase (CAT) and SOD, while its administration in Ovx sheep decreases SOD, CAT and GPx^9^.

Oophorosalpingohysterectomy (OSH) consists of the surgical removal of the uterus and the ovaries in their entirety. In addition to changing the hormonal profile, this procedure influences aspects such as longevity which is reduced; it produces behavioral changes, induces overweight and obesity and increases the incidence of specific diseases^11^. It has been described that the OSH along with physical inactivity and a spontaneous increase in food intake constitute risk factors for female dogs to develop overweight and obesity^12^. This is the reason why calorie restriction is recommended to avoid weight gain in pets that have undergone OSH^12^.

In previous studies it has been observed that after the performance of OSH there is an increase in lipogenesis and OS^9–11^. However, studies on the evaluation of antioxidant/oxidant status related to the absence of 17β-E_2_ in female dogs with OSH are limited. Therefore, the goal of this study was to evaluate the changes in some serum antioxidant enzymes one and six months after surgery and in the morphology of subcutaneous adipocytes before and six months after OSH in young female dogs.

## Results

### General characteristics

**Figure 1:**
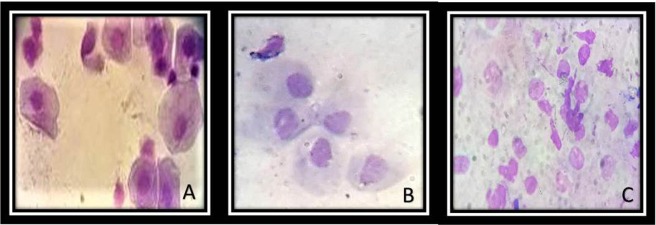
Representative photomicrographs 100x Exfoliative vaginal cytology basal, first and sixth month respectively. Representative photomicrographs in basal conditions (**A**), first (**B**) and sixth (**C**) month after OSH. The cytologies show basal cells of the anestrus stage; this stage is the time between diestrus and the next proestrus and it’s recommended for the realization of the OSH, (n = 12).

Weight at baseline, the %BF and body condition (BC) tended to increase six months after OSH in each of the female dogs without there being statistically significant changes (Table 1). The general characteristics of the biochemical serum tests in the experimental groups are shown in Table 2. The variables that did not show a significant difference in the first and sixth months after performing the OSH were: cholesterol, triglycerides and glucose. Insulin and the HOMA index were increased in the first month after OSH with a significant difference (p = 0.01). 17β-E_2_ showed no significant difference, however, a tendency to decrease was observed in the first and sixth months after OSH. The opposite effect was observed in serum T concentrations which showed a significant increase. Figure 1 shows representative photomicrographs of the exfoliative vaginal cytology in the basal conditions (A), and one (B) and six (C) months after surgery respectively. The cytologies show basal cells characteristic of the anestrus stage; this stage is the time between diestrus and the next proestrus and is the recommended time for the performance of the OSH.

**Table 1:** General characters of the female dogs in basal conditions, 1 and 6 months.

Dog breed	Age	Pet food	Feed method	Basal			1 month			6 month		
				Weight (kg)	%BF	BC	Weight (kg)	%BF	BC	Weight (kg)	%BF	BC
*Pug*	3	Ganador	Portions	6.40	27.06	4	6.30	27.06	4	7.20	28.31	4
*Pug*	2	Ganador	Portions	8.00	24.43	3	8.30	25.36	4	10.00	28.63	4
*Schnauzer*	1	Dog Chow	Portions	7.20	17.31	3	7.60	19.01	3	8.80	25.36	3
*Mixed-breed*	4	Nupec	Portions	5.00	18.08	3	5.20	19.62	3	5.60	23.34	3
*Mixed-breed*	2	Dog Chow	Portions	12.00	19.33	3	12.50	19.33	3	12.50	22.28	3
*Mixed-breed*	2	Óptimo	Portions	13.00	22.44	3	13.10	22.44	3	13.90	23.37	3
*Mixed-breed*	3	Óptimo	Portions	16.80	22.12	3	17.50	20.58	3	18.20	19.04	3
*Dalmatian*	5	Dog Chow	Portions	22.00	22.60	3	21.90	23.53	3	20.90	19.97	3
*Pitbull*	2	Benefult	Portions	21.50	23.69	3	22.40	24.62	3	27.60	28.34	4
*Pitbull*	5	Dog Chow	Portions	24.80	24.78	3	25.50	30.98	4	28.00	31.45	4
*Boxer*	3	Óptimo	Portions	31.60	25.10	3	32.00	30.36	4	39.50	32.38	4
*Dogue of Bordeaux*	4	Campeón	Portions	42.60	25.58	3	50.00	43.09	5	61.50	41.55	5

Abbreviations: BC = Body condition, %BF = Body fat.

**Table 2:** General characteristics of blood chemistry and concentration of sex hormones in female dogs in basal conditions, 1 and 6 months.

Variables	Basal	1 month	6 month
Cholesterol (mg/dL)	233.16 ± 35.31	190.25 ± 17.03	186.66 ± 15.57
Triglycerides (mg/dL)	51.16 ± 13.91	61.50 ± 8.40	52.50 ± 6.94
Glucose (mg/dL)	94.66 ± 8.87	91.33 ± 2.88	85.16 ± 10.51
Insulin (µU/mL)	6.96 ± 1.89	**16**.**42 ± 2**.79*	8.05 ± 1.12
HOMA-IR	1.66 ± 0.47	**3**.**83 ± 0**.82*	1.68 ± 0.31
Estradiol (pg/mL)	69.70 ± 29.87	41.73 ± 2.13	40.78 ± 1.24
Testosterone (ng/mL)	0.44 ± 0.17	0.57 ± 0.21	0.79 ± 0.39

Values expressed in mean ± standard error, (n = 12). *Basal vs. 1 month p = 0.01.

### Cell count by field and adipocyte area in the subcutaneous adipose tissue

**Figure 2:**
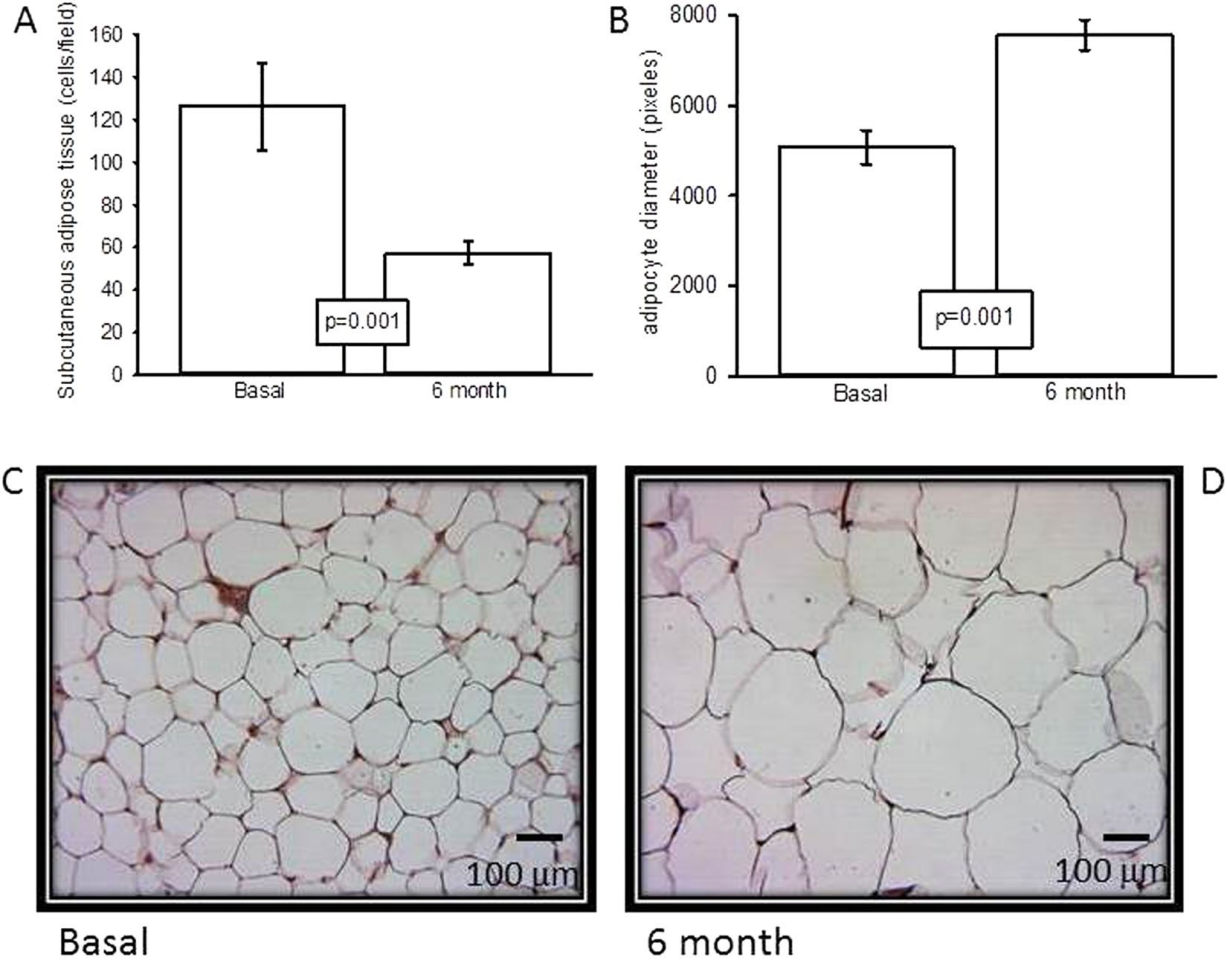
(**A**) Subcutaneous adipose tissue in basal conditions and six months after OSH. (**B**) Adipocytes diameter in basal conditions and six months after OSH. Values expressed in mean ± standard error, (n = 12). And representative photomicrographs of subcutaneous adipose tissue in (**C**) basal conditions and D) six months after OSH.

**Figure 3:**
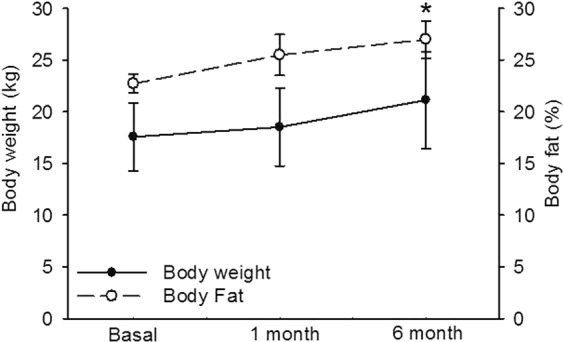
Body weight and fat in dogs females in basal conditions, first and sixth month after performing the OSH. Basal conditions vs. after six months (*p = 0.04). Values expressed as mean ± standard error (n = 12).

Figure 2A shows that, the number of cells per field six months after OSH is significantly lower (p = 0.001) than the basal values. At the same time, adipocyte diameter in the samples taken six months after the OSH is significantly larger (p = 0.001) than previous to the surgery (Fig. 2B). In addition, Fig. 2C,D show representative photomicrographs of white adipose tissue, Fig. 2C corresponds to the basal condition. In it, polyhedral cells are found, with a lipid drop that fills the entire cytoplasm and displaces the nucleus towards the periphery. A greater number of cells are observed when compared to Fig. 2D, which corresponds to the sample taken six months after the OSH. The cells are arranged in irregular hexagonal groups and connected by cleft unions when compared to those found in the basal conditions. Hypertrophy of the adipocytes is also observed. Figure 3 shows that body fat was significantly increased in the sixth month in comparison with the basal condition (p = 0.04). The same figure shows that there was not a significant difference in body weight between the groups of the first and sixth months with respect to the basal condition.

### Extracellular SOD and glutathione employing enzymes

**Figure 4:**
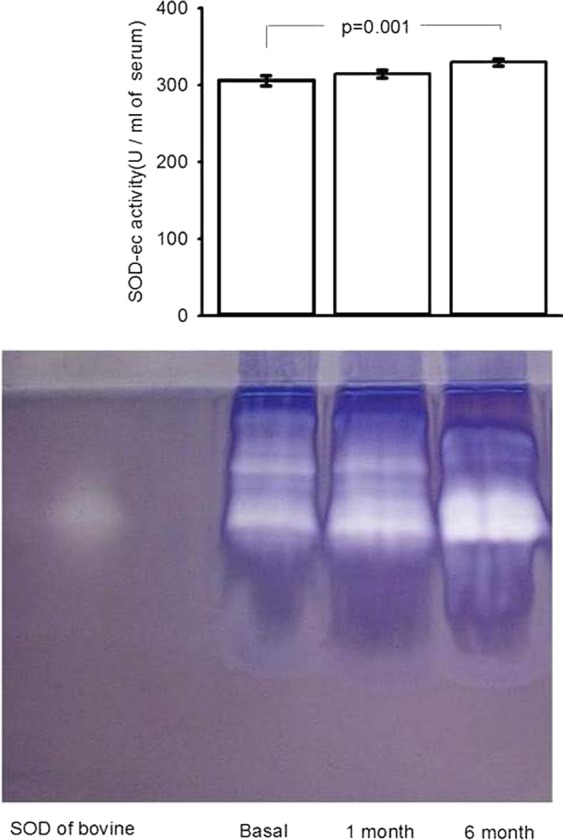
SOD-ec expression in basal conditions, first and sixth month after of the OSH. Values expressed in mean ± se. (n = 12).

**Figure 5:**
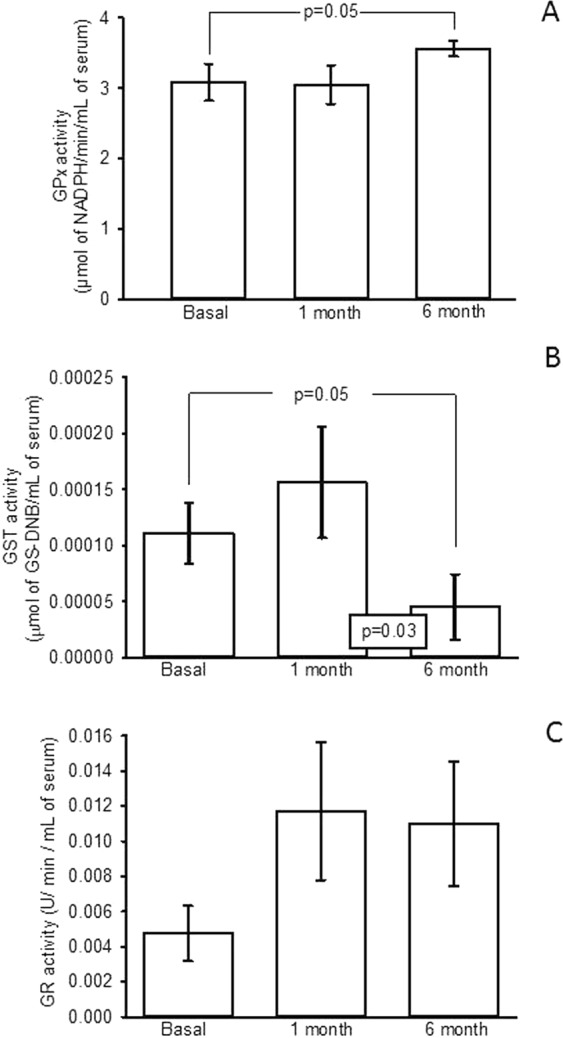
(**A**) Enzymatic activity of the GPx, (**B**) Enzymatic activity of the GST and (**C**) Enzymatic activity of the GR The experimental conditions were in basal conditions, first and sixth after OSH. Values expressed in mean ± standard error, (n = 12).

Figure 4 show that, the SOD-ec activity in basal conditions and after the first month did not show a statistically significant difference. However, six months after OSH, there was a significant increase in the activity when compared to the baseline condition (p = 0.001). The GPx activity was significantly increased six months after the surgery when compared to the baseline condition (p = 0.05). There was no difference between the baseline condition and the first month after OSH (Fig. 5A). The GST activity did not show a significant difference between the baseline condition and the first month after OSH. However, a significant increase was observed between the basal conditions and the measurement six months after the procedure. There was also a significant difference between the first and sixth month after OSH (p = 0.05, p = 0.03 respectively, Fig. 5B). In addition, the GR activity showed no significant difference between the groups of the first and sixth months with respect to the basal condition. However, there was a tendency to increase when compared to the basal condition (Fig. 5C).

### Markers of oxidative stress

**Figure 6:**
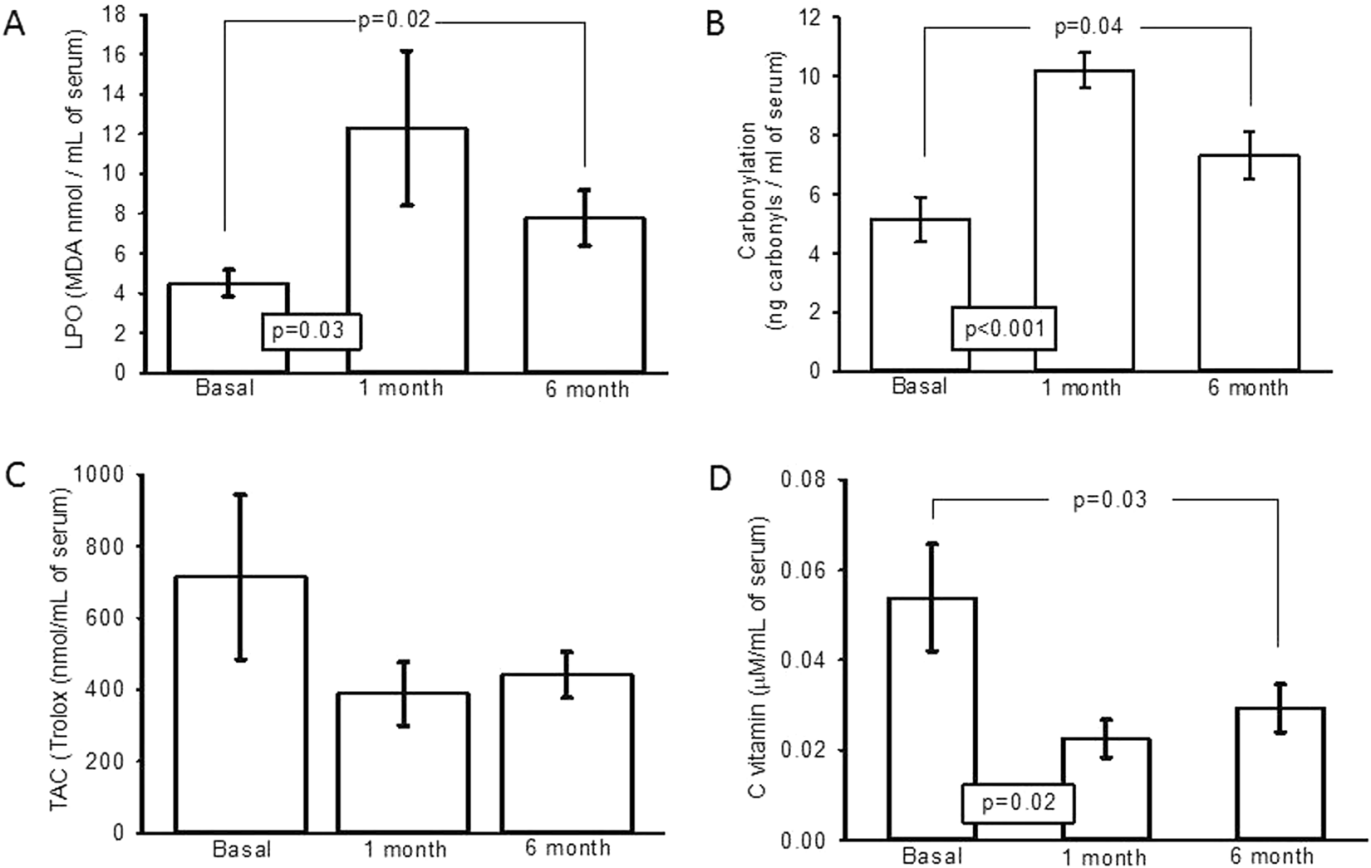
(**A**) Lipid peroxidation (**B**) Carbonylation, (**C**) Total antioxidant capacity and (**D**) Vitamin C. The experimental conditions were in basal conditions, first and sixth month after OSH. Values expressed in mean ± standard error; (n = 12).

LPO showed a significant increase in the first and sixth month after OSH with respect to the basal conditions (p = 0.03, p = 0.02 respectively, Fig. 6A). Carbonylation showed a significant increase in the first and sixth month after OSH with respect to the basal conditions (p < 0.001, p = 0.04 respectively, Fig. 6B). Figure 6C shows that the TAC was not significantly different between the groups of the first and sixth month when compared to the basal conditions; however, a tendency to decrease when compared to the baseline conditions was observed. In Fig. 6D, a significant decrease in vitamin C between the first and sixth month after OSH with respect to the basal conditions can be observed (p = 0.02, p = 0.03, respectively).

### GSH and GSSG concentration and GSH/GSSG index

**Figure 7:**
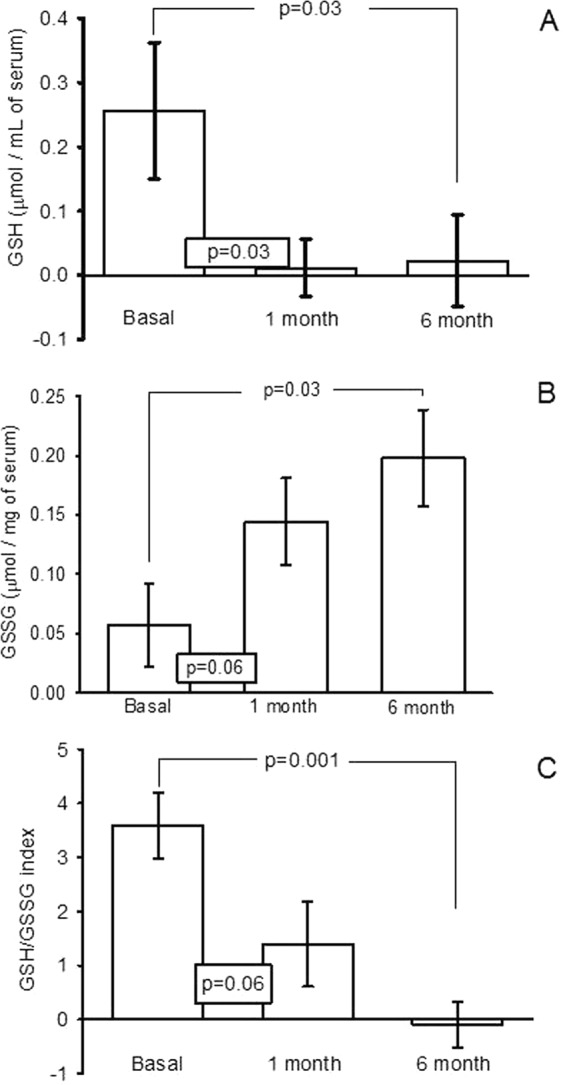
Concentration in serum of the: (**A**) GSH, (**B**) GSSG, and (**C**) GSH/GSSG index in basal, first and sex month after of OSH. Values expressed in mean ± standard error (n = 12).

Figure 7A shows that the concentration of GSH was significantly decreased in the first and sixth month, when compared to the basal condition (p = 0.03). Figure 7B shows the GSSG concentration which was significantly increased in the first and sixth month in comparison with the basal condition (p = 0.03). Figure 7C shows the GSH/GSSG index which was significantly decreased in the sixth month in comparison with the basal condition (p = 0.001).

## Discussion

17β-E_2_ possesses a great variety of functions in female organisms, among which the regulation of food consumption, of the estrous cycle, of fat corporal distribution and of the antioxidant function can be mentioned^13^ Nevertheless, 17β-E_2,_ at high concentration, may induce development of cell damage by OS through the metabolic reactions of its phenolic ring^4^. When it is found in high concentrations, the development of OS might become its predominant biochemical activity and it could exert deleterious effects^7^. When OSH is performed, female dogs exhibit changes that might be a consequence of reduced plasma concentration of this hormone. It has been described that female dogs that underwent OSH show an increase in body weight, %BF and OS index^14^ among other effects. Therefore, the aim of this study was to determine the impact of OSH on the antioxidant system, as well as to quantify changes in the morphology of subcutaneous adipocytes in female dogs.

Our results show that after OSH, the serum concentrations of 17β-E_2_ tended to decrease but did not completely disappear, despite the proper OSH performance. The surgery’s correct performance was proven by the presence of cells in anestrus in the vaginal exudate after the first and second month of surgery (Fig. 1A–C) and by the increase in the weight of the dogs. In a study in female dogs where a partial OSH was performed, with elimination of only one uterine horn, the 17β-E_2_ levels did not vary from the beginning of the surgery to six months after the procedure was performed. This previous study suggested the need of a greater period than six months for the total elimination of the hormone, and the authors suggested that the presence of 17β-E_2_ could be attributed to the amount of adipose tissue^15^, to the production of hormone by the adrenal gland and/or to external factors such as food consumption^1^. The incomplete disappearance of the hormone in our study could be due to the ingestion of the phytoestrogens in the diet. Most pet foods use soy or soybean fractions as ingredients^16^. These components of the diet are a source of phytoestrogens that can have both beneficial and harmful effects when chronically consumed^16^. In addition, phytoestrogens are structurally and functionally similar to 17β-E_2_ and have the ability to bind to the 17β-E_2_ receptors (ER), interacting more with ER-α than with ER-β^17^. Phytoestrogens may compete for the active binding site of 17β-E_2_ and can contribute to maintain the profile of the hormone^17^. Furthermore; these molecules can contribute to the regulation of the expression of certain antioxidant enzymes through NrF2^18^. However, more studies are required to elucidate this issue. Although in the present study we did not observe a significant difference in the level of the hormone, there is a tendency of 17β-E_2_ to decrease and the T concentration growed gradually until six months after the OSH. Several studies have shown that T may stimulate lipogenesis and its levels have been associated with adipocyte hypertrophy^19^.

Our results show that female dogs gain weight and there is an elevation %BF which is reflected in the photomicrographs after OSH. Prior to OSH, a large number of adipocytes comprising a reduced area were observed, while six months after surgery, the adipocytes show a much larger area and are found in a smaller amount. This result suggests that 17β-E_2_ can contribute to the regulation of the distribution, size and number of the adipose tissue cells as has been previously reported^7^. This effect is contrary to that of T^7,20^. The removal of 17β-E_2_ by OSH favors an increase in subcutaneous fat, which is characterized by large adipocytes. In a study in female dogs, 17β-E_2_ had a direct effect on the satiety center (hypothalamus) and this may indirectly influence the hormonal regulators of food intake (ghrelin and leptin) affecting weight gain and obesity^21^. The absence of 17β-E_2_ increases the probability of developing overweight or obesity^22^.

Furthermore, most pet owners offer their pets food without considering the energy and nutritional contribution provided, which in many cases proves to be excessive^23^. Some components of commercial pet foods, such as lipids (fatty acids FA) and soy flour, are used to improve their palatability, but in excess, these FA may accumulate in adipose tissue in the form of TG, causing an increase in adipose tissue^24^.

The accumulation of visceral fat is associated with low levels of 17β-E_2_ in females. Some studies with animal models have observed that 17β-E_2_ may act as an anorexigenic hormone increasing energy expenditure at rest^25^. In addition, 17β-E_2_ participates in energy consumption due to its direct effects on peripheral adipose tissue, increasing the number of its receptors, the sensitivity to leptin and thermogenesis through actions on the central nervous system. Consequently, this hormone increases lipolysis in the white adipose tissue (WAT)^26^. In rodents, 17β-E_2_ reduction due to OSH may cause hyperphagia and increases in body weight. However, with the replacement of this hormone, the adipocyte size decreases, and there is inhibition of lipogenesis in WAT, in the liver, in skeletal muscle and an increase of lipolysis in the adipocytes^27^. These changes can be attributed to the transcription and gene expression of proteins that control the storage of lipids such as acetyl-CoA carboxylase 1, fatty acid synthase, sterol regulator of the protein-1 binding element, and to the lipoprotein lipase (LPL) suppression^7^. There is also an increased sensitivity to lipolysis induced by catecholamines in the adipose tissue. Other studies mention the suppression of LPL transcription, as another role of the hormone^27^. This enzyme catalyzes the conversion of TG into free FA^11^. In addition, adipose tissue produces a wide variety of bioactive molecules including TNFα, IL-6, adiponectin, leptin, among others^5^. Therefore, when overweight or obesity is present, adipose tissue promotes the synthesis of these pro-inflammatory cytokines, which cause an increase in ROS^28^.

The increase in the amount of adipose tissue is related to the presence of insulin resistance (IR)^29^. Therefore, we determined the HOMA-IR index in our study. Our results suggest that the tendency to decreased 17β-E_2_ could lead to a period of IR in the first month after OSH and that this condition might disappear after a longer period. This could be a long-term risk factor that could be associated with diseases such as diabetes, obesity and hypertension^4^. However, six months after the surgery, the HOMA-IR index decreased almost to the basal value suggesting that the condition of IR might be temporary and could disappear after a longer time period. Since there was not a clear tendency of the HOMA-IR index to increase in this study, determinations after more than six months would be necessary to prove this hypothesis.

17β-E_2_ regulates the nuclear type 2 pathway (Nrf2) that constitutes an essential transcription factor for the antioxidant defense mechanism. This pathway participates in the induction of the transcription of some genes and antioxidant enzymes that act synergistically to eliminate the ROS^30^. A study in mice showed that Nrf2 is involved in the signaling of the transcription of antioxidant enzymes such as SOD, GST, GPx and GR^29^.

The first line of defense against O_2_^−^ is SOD, which is responsible for detoxifying O_2_^−^ in H_2_O_2_^31^. This antioxidant enzyme can protect cells from the potentially harmful effects of ROS. SOD-ec is an antioxidant enzyme present in extracellular fluids such as serum^4^ and is primarily expressed in blood vessels on the surface of vascular smooth muscle cells and the sub-endothelial space. The results of this study suggest that OSH may increase O_2_^−^ and favor the activity of SOD-ec despite the tendency of 17β-E_2_ to decline. Consequently, H_2_O_2_ is increased. The SOD-ec expression and activity can be altered in response to a variety of stimuli including hypertension, diabetes, and ROS^4^. Our results show that SOD-ec activity is significantly increased six months after the OSH. This suggests that OSH in female dogs can increase SOD-ec activity, reducing O_2_^−^ but increasing H_2_O_2_ in serum. The increase of this reactive species may contribute to LPO and carbonylation.

Although these results can seem paradoxical, it has been reported that physiological concentrations of 17β-E_2_ may lower H_2_O_2_ levels through the MAPK and NF*κ*B pathways that modify the expression of SOD isoforms^8^. 17β-E_2_ can also modulate the Nrf2 pathway, which controls the expression and induction of genes that encode for SOD isoforms^32^. It would be expected that the decrease in 17β-E_2_ would decrease the SOD-ec expression or activity, however this was not observed and the activity of SOD-ec was increased instead. The explanation for this discrepancy could be due to: 1) phytoestrogens provided by the diet that could favor/contribute to the expression or activity of this enzyme, 2) OSH could favor the increase of the O_2_^−^ that is the substrate of the SOD-ec, and this may raise its activity. Furthermore, treatment with 17β-E_2_ normalized the SOD activity in liver fractions from 24-month-old menopausal female rats^33^.

Detoxification of H_2_O_2_ occurs mainly through the catalase and GPx pathways. It has been described that 12% of the SOD-ec functions are related to the activity of some of the GPx isoforms^34^. In addition, GPx transcription can be regulated directly or indirectly by Nrf2 through the action of 17β-E_2_^35^. Our results show that the GPx activity was increased in female dogs after of OSH. These results could also be seen as paradoxical since it has been reported that in murine skeletal muscle and adipose tissue, the genes that encode for GPx are sensitive to 17β-E_2_^36^. Another study described that GPx activity was significantly higher in premenopausal women than in women after menopause^37^. The ability of 17β-E_2_ to regulate GPx transcription may contribute to increase of the expression or activity of the GPx in females^38^, and this, in part, can contribute to reduce lipid hydrogen peroxides, and decrease LPO. However, our results suggest that the phytoestrogens provided by the diet could prevent the net loss of serum 17β-E_2_, and thus contribute to restore the activity of this enzyme^39^. However, more studies are needed to elucidate this mechanism.

For H_2_O_2_ to be reduced to water by GPx, the enzyme requires of GSH as electron donor, which is then oxidized to GSSG. GSSG is later reduced again to GSH by means of the NADPH-dependent GR^40^. These components of the antioxidant system interact as a feedback system^41^ and GSH concentration depends on the equilibrium between its consumption and its biosynthesis which can be analyzed by the GSH/GSSG index^42^.

Our results show that the GSH depletion and the GSSG elevation can be a consequence of the decrease of the non-enzymatic antioxidant system and the increase in the activities of the GPx. The tendency to an increase in GR activity observed, suggests that its activity is insufficient to reestablish the concentrations of the exhausted GSH. GR is the enzyme responsible for the regeneration of GSH. Furthermore, the activity of GR is important to control the level of GSSG. The uncontrolled generation of GSSG during OS, may limit the activity the GPx and GST enzymes that depend on GSH. Furthermore, GSSG can also accumulate inside the cell and react with the sulfhydryl groups of proteins to produce GSH-disulfide proteins, which have longer half-lives and reduce the amount of poorly folded proteins^4^ which contribute to increase of protein carbonylation.

Therefore, our results suggest that the 17β-E_2_ and T changes can control GR activity and GSH/GSSG ratio. However, another possible explanation is that the synthesis of GSH could be influenced by the tendency of 17β-E_2_ to be reduced. Under normal conditions, there is a synergic effect between the hormone and GSH that protects against the OS^43^. The relationship between GSH and 17β-E_2_ is attributed to Nrf2, which regulates the antioxidant response through the transcription of gamma-glutamyl cysteine ligase involved in the GSH synthesis^42^.

Another enzyme that participates in ROS detoxification is GST which prevents cells from the effects of LPO. This enzyme conjugates GSH to electrophilic agents, forming a bond and detoxifying them. 17β-E_2_ modulates the expression and induction of genes that encode for GST^22^. GSTA4 expression is diminished in obese insulin-resistant C57BL/6J mice and in humans with obesity-linked IR^32^. In addition GST is intimately involved in the biosynthesis of T. However, the effect of the concentration of these hormones on their activity is unknown^44^. Our results suggest that OSH decreases GST activity in dogs which is associated with a tendency to decrease 17β-E_2_ and increase T. OSH alters the activity of this enzyme contributing to the increase in LPO, carbonylation and reduced TAC.

Therefore, our results show that the antioxidant enzymes have adaptive characteristics altering their activity when 17β-E_2_ is reduced. This, in turn, can increase the production of ROS^45^. The results obtained one month after the OSH, might be interpreted as the consequence of the process of stabilization in the organism.

Vitamin C is another component used in pet foods that participates in the antioxidant system and is also considered as a protector against the FA that the food contains^4^. Vitamin C inhibits the production O_2_^−^ and peroxynitrite by decreasing NAPH oxidase that produces O_2_^−^ and the inducible nitric oxide expression^4^. Dogs can also synthesize this component. Consequently, this vitamin is found constantly in the body^46^. Our results show that the vitamin C concentration decreased after OSH. This could be attributed to its relationship with GSH which participates in the regeneration of the active form of vitamin E. GSH levels could cause regeneration of vitamin C^3^. Furthermore, vitamin C can be preserved by melatonin (MEL)^47^. In this regard, it has been described that MEL possesses powerful antioxidant effects by decreasing oxidative damage at the cellular and intracellular membrane level, due to its hydrophilic and lipophilic properties. It also functions as a terminal antioxidant molecule since once bound to free radicals it metabolizes it into terminal products that are eliminated in the urine^47^ and can attenuate LPO and carbonylation. 17β-E_2_ deficiency is associated with reduced MEL secretion and this plays an important role in the weight gain process^48^. This suggests that MEL supplementation could have a beneficial effect on the body weight reduction and OS after of OSH^49^.

LPO and carbonylation are the result of the alteration of the enzymatic and non-enzymatic oxidative system, and therefore, both are considered as markers of the damage generated by the ROS. ROS commonly attack the unsaturated lipids that make up the cell membrane^8^, but also attack proteins modifying side chain amino acid groups. 17β-E_2_ can reduce LPO and carbonylation, and its reduction might generate the opposite effect on stress markers like LPO, carbonylation and TAC^50^. In the present study, we found that LPO, carbonylation and TAC showed an increase and tendency to decrease respectively after OSH, due to changes in physiological concentrations of 17β-E_2_^51^. Another study conducted in female dogs showed that after OSH, there is a period of adaptation and imbalance in the antioxidant-oxidant system, in which there was an increase in the SOD, LPO, carbonylation and a decrease in the GSH^52^. These results are in agreement with those found in our study.

In conclusion, in this paper we found that OSH tends to decrease serum levels of 17β-E_2_. This decrease contributes to increase body weight and causes hypertrophy of subcutaneous adipose tissue. The surgery also alters the activity of the oxidant-antioxidant system which, in turn, increases LPO and carbonylation. Thus, there is a deterioration of the antioxidant state. However, the diet phytoestrogens and food enriched with antioxidant supplements could alter the activity of the antioxidant enzymes and might be beneficial for the health of sterilized female dogs by OSH.

### Study limitations

It is difficult to carry out studies when a high affective value for pets (female dogs) by their owners is present. Owners only agreed to a period of time for the study that did not exceed 6 months, and therefore it was only possible to obtain two samples of adipose tissue; one at the beginning and the other at the end of the study. The obtainment of tissue samples is considered as an invasive method due to the necessary use of anesthesia. Other limitation this study could be the OSH surgery, which can increase OS per se in comparison with laparoscopic surgery^14^, however this would be during the intervention and some days after the surgery, the evaluation time of this study may reduce this and reflect only the change in the antioxidant status altered by OSH and the 17β-E_2_ decrease.

## Methods

### Ethics statement

Experiments in female dogs were approved by the Laboratory Animal Care Committee of our institution with protocol number MC-2017/2-13 and were conducted in compliance with the Guide for the Care and Use of Laboratory Animals.

### Animals

12 Female dogs between 1 to 5 years of age were used with authorization from the owners, Information such as: age, race, type of feeding, weight, body condition, pelvic circumference (PC), distance from the knee to the hock (KH) and amount of physical activity was collected for each individual. Inclusion criteria: Whole more than 12 months of age (1 to 5 years old) animals, (with ovaries and uterus) that were clinically healthy were accepted. Animals had a body condition of three and were of any size. Exclusion criteria: The presence of achondroplasia, canine females that were within group 10 of the hounds according to the international canopy federation, males and animals with the presence of a clinical disease were excluded.

An initial blood sample was taken to measure the basal activities of antioxidant enzymes and hormones such as 17β-E_2_, insulin and T. Before the surgery, the stage of the estrous cycle in each individual was determined by means of vaginal exfoliative cytology and then the OSH procedure was performed. One and six months after the surgery, blood samples and vaginal cytology were repeated.

### Morphometric data, weight and body condition

At the time of measurement, the dogs were placed in a quadripedestation position with the head facing the front, forming a right angle. The PC and the distance that exists from the KH were measured with a metric tape (cm) without exerting any type of pressure on the female dog.

The weight was obtained with a digital scale (brand: SohenleTM) and the body condition was determined based on the percentage of body fat (%BF) resulting from the HAND formula^53^: %BF = −1.7(LM _(cm)_ + 0.93(PC _(cm)_) + 5. Where; LM = Length of the hind limb from the calcaneal tuberosity to the middle of the patellar ligament (KH) in cm. PC = pelvic circumference in cm. Sample size was calculated by formula of the specific difference between groups.

$$\frac{{\rm{n}}=4{{\sigma }}^{2}{({{Z}}_{1-{\boldsymbol{\alpha }}/2}+{{Z}}_{1-{\boldsymbol{\beta }}})}^{2}}{{({\mu }1-{\mu }2)}^{2}}$$

Where: n = sample number, α = Probability of type 1 error, β = Probability of type 2 error, σ = standard deviation, µ1 = Expected average of the first group and µ2 = Expected average of the second group, Z = area under the curve with normal distribution.

### Measurements in the blood sample

The blood sample was obtained by venipuncture of the cephalic vein (previous asepsis) and blood was placed in tubes without anticoagulant. It was allowed to stand for a period of 15 minutes and centrifuged at 3000 rpm. Finally, the serum was recovered and frozen at −6 °C. Triglycerides (TG), cholesterol (CT), glucose and insulin were determined in the serum by the ELISA technique using commercial kits. Serum 17β-E_2_ and T were measured by RIA using the Diagnostic Products Corporation Kits (Los Angeles, CA), and following the routine method recommended by the manufacturer. The insulin resistance index (HOMA-IR) was calculated with the following formula: HOMA-IR = [insulin (μU / ml)*glucose (mM)]/22.5

### Lateral OSH

The initial incision was made in the right flank because the ligament of the left ovary is slightly longer and lax, which allows for the extraction of this ovary by the opposite flank with ease. The points taken as reference were the limit of the muscular portion of the external abdominal oblique, the dorsal projection of the nipple, the edge of the transverse processes of the lumbar vertebrae, the anterior edge of the pubis and the last rib. An incision of approximately 1–2 cm (depending on the size of the bitch) was made on the line that starts from the angle of the joint of the last rib and ends at the anterior edge of the pubis. After the incision in the skin, the muscle fibers were separated and once reaching the abdominal cavity, the Farabeuf spacers were placed to visualize the right ovary and the respective uterine horn which were going to be extracted together with their vascularization. This package was clamped, ligated and cut at the level of the insertion of the suspensory ligament of the ovary. Then, a light traction was performed to remove the body from the uterus which was pinched, ligated and cut behind the cervix (to avoid post-infection). Finally, the uterine horn was extracted with its respective ovary on the left side to perform the same procedure with the right ovary. The peritoneum was closed with a “U” point and the subcutaneous tissue and skin were sutured with separate simple points^54^.

### Extra cellular superoxide dismutase activity

SOD-ec enzyme activity was determined in serum by non-denaturing gel electrophoresis and nitro blue tetrazolium (NBT) staining^9^. 20 μl of serum were applied directly, without boiling, to a non-denaturing 10% polyacrylamide gel. The electrophoresis was carried out at 120 volts for 4 hours. Subsequently, the gel was incubated in a 2.45 mM NBT solution for 20 min, then the liquid was discarded and the gel was incubated in a 0.028 mM EDTA solution, containing 36 mM potassium phosphate (pH 7.8) and 0.0028 mM riboflavin. SOD-ec activity was expressed in U/ml of serum. Purified SOD from bovine erythrocytes, with a specific activity of 56 U/µg of protein (Sigma-Aldrich, St. Louis, MO, USA). The gels of SOD-ec were analyzed by densitometry by the image analyzer Sigma Scan Pro5.

### Glutathione peroxidase

For GPx activity, 100 μl of serum were suspended in 1.6 ml of 50 mM phosphate buffer (pH 7.3), with added 0.2 mM NADPH, 1 mM GSH and 1 UI/ml GR. The mixture was incubated for 3 minutes at 37 °C, then 100 μl of 0.25 mM H_2_O_2_ were added to start the reaction and the absorbance was monitored for 10 min at 340 nm. Activity is expressed in µmol of NADPH oxidized/min/ml serum^8^.

### Gluthatione-S-transferase

The activity of GST was determined spectrophotometrically, 700 μl of phosphate buffer (0.1 M, pH 6.5) supplemented with 100 µl GSH 0.1 mM and 100 µl 1-Chloro-2, 4-dinitrobenzene (CDNB) 0.1 mM was added to 100 µl of serum. The sample was incubated and monitored for 10 min at 37 °C at 340 nm. Values of GST activity were expressed in U/min/ml of serum. A unit of activity of GST is expressed in µmol of GS-DNB conjugate formed/min/ml serum at 37 °C^8^.

### Glutathione reductase

To evaluate GR activity, 700 μL of phosphate buffer 0.2 mM, plus 0.5 mM of EDTA pH 7.3, 100 μl de NADPH 0.1 mM, and 100 μl of serum were mixed. Then they were incubated and monitored for 10 min at 37 °C and the absorbance was read at 340 nm^43^. GR activity is expressed in U/min/ml of serum. A GR unit is defined as the amount of enzyme needed to reduce one µmol of GSSG per minute.

### GSH and GSSG concentration

To determine GSH concentration, 800 μl of phosphate buffer 50 mM, pH 7.3, plus 100 μL of Ellman reactive (5, 5′ dithiobis 2-nitrobenzoic) 1 M were added to 100 μl of serum previously deproteinized with 20% trichloroacetic acid (vol/vol) and centrifugated to 10000 rpm for 5 minutes. The mixture was incubated at room temperature for 5 minutes and absorbance was read at 412 nm. The calibration curve was done with GSH at concentrations from 0.15 to 10 μmol^43^. To determine GSSG concentration, 100 μl of serum previously deproteinized with 20% trichloroacetic acid (vol/vol) and centrifugated to 10000 rpm for 5 min. The supernatant was recovered and added 8 μl of 4-vinylpiridine, incubated 5 min. and added 400 μl of KH_2_PO_4_ 50 mM, pH 7.3, plus 100 μL of Ellman reactive 1 M, incubated at room temperature for 5 min. The calibration curve was made with GSSG at concentrations from 0.15 to 10 μmol/ml, the absorbance was read at 412 nm^43^.

### Lipid peroxidation

50 μl CH_3_-OH with 4% BHT plus phosphate buffer pH 7.4 were added to 50 μl of serum. The mixture was shaken vigorously in vortex for 5 seconds and then incubated in a water bath at 37 °C for 30 min. 1.5 ml of 0.8 M thiobarbituric acid were then added and the sample was incubated in a water bath at boiling temperature for 1 hour. After this time and to stop the reaction, the samples were placed on ice; 1 ml 5% KCl was added to each sample as well as 4 ml n-butanol; they were shaken in vortex for 30 seconds and centrifuged at 4000 rpm at room temperature for 2 min. Then the n-butanol phase was extracted and the absorbance was measured at 532 nm. The calibration curve was obtained using tetraethoxypropane as standard^8^.

### Evaluation of total antioxidant capacity

50 μl of serum ware suspended in 1.5 ml of a reaction mixture prepared as followed: 300 mM acetate buffer pH 3.6, 20 mM hexahydrate of ferric chloride, 10 mM of 2,4,6-Tris-2-pyridil-s-triazine dissolved in 40 mM chlorhydric acid were added in a relation of 10:1:1 v/v respectively. The mixture was shaken vigorously in a vortex for 5 seconds. It was then incubated at 37 °C for 15 min in the dark. The absorbance was measured at 593 nm. The calibration curve was obtained using Trolox^18^.

### Vitamin C

20% trichloroacetic acid was added to 100 µl of serum. After vigorous shaking the samples were kept in an ice bath for 5 min and centrifuged at 5000 rpm for 5 min, 200 µl of Folin-Ciocalteu reagent 0.20 mM was added to the supernatant. The mixture was shaken vigorously in a vortex for 5 seconds and incubated for 10 min. The absorbance was measured at 760 nm. The calibration curve was obtained using ascorbic acid standard solution^18^.

### Carbonylation

100 μl of serum were added to 500 μl of HCl 2.5 N in parallel other sample with 500 μl of 2, 4-dinitrophenylhydrazine (DNPH), and incubated in the dark at room temperature for one hour, shaking with a vortex every 15 min. At the end of the incubation period, 500 μl of 20% trichloroacetic acid was added, and centrifuged at 15,000 g for 5 min. The supernatant was discarded. Two washings were performed, first removing the precipitate with a sealed capillary tube, adding 1 ml ethanol/ethyl acetate was incubated by 10 min, and centrifuging at 15000 g for 10 min. Finally, 1 ml of 6 M guanidine hydrochloride in 20 mM KH_2_PO_4_ pH 2.3 was added. The mixture was incubated again at 37 °C for 30 min. Absorbance was read in a spectrophotometer at 370 nm, using water bi-distilled as blank and a molar absorption coefficient of 22000 M^−1^ cm^−1^.

### Adipose tissue

After lateral OSH and six months after this procedure, in previously anesthetized animals with the use of Procin equus: Xylazine 10% at a dose of 1.1 mg/kg IV [Intravenous] and Zoletil® 100: Tiletamine/Zolazepam at a dose of 2 mg/kg IV), a sample of approximately 1 cm of adipose tissue was taken (right flank in the same anatomical site of the OSH). The subcutaneous tissue and skin were sutured with separate simple points (Atramat suture: polyglycolic acid). Antibiotic was finally applied (Espenfort: Procaine penicillin G at a dose of 20,000 IU/kg intramuscular) and analgesic (Angesin: Dipyrone sodium at a dose of 28 mg/kg intramuscular) to female dogs in order to avoid any discomfort.

### Subcutaneous adipose tissue histology

For histology, 2 mm of subcutaneous fat were washed in 0.9% NaCl for 30 sec. The solution was then decanted and phosphate buffer with 10% formalin was added for 24 hours. The histological sections were processed according to conventional histological procedures and stained with Masson technique. Histological sections were analyzed using a light microscope Carl Zeiss (63300 model) equipped with a Tucsen (9 megapixels) digital camera with software TSview 7.1, at a 40x magnification. The photomicrographs were analyzed by densitometry using Sigma Scan Pro 5 Image Analysis software. The density values are expressed as pixel units.

### Statistical analysis

To determine the size of the sample, the Win Episcope® program was used, with a confidence level of 95% with one-way paired samples. Statistical analysis and graphics were performed with the Sigma Plot 12.3 program version, Jendel Corporation, 1986–2012. The data are presented as the mean ± SE. Statistical significance was determined by one-way ANOVA test, followed by Tukey’s post hoc test. Differences were considered as statistically significant at p < 0.05.

## Data Availability

The datasets generated and analyzed during the current study are available from the corresponding author on reasonable request.
